# The relationship between clinical presentation and the nature of care in adults with intellectual disability and epilepsy – national comparative cohort study

**DOI:** 10.1192/bjo.2024.45

**Published:** 2024-04-30

**Authors:** Sarah Badger, Lance V Watkins, Paul Bassett, Ashok Roy, Mogbeyiteren Eyeoyibo, Indermeet Sawhney, Kiran Purandare, Laurie Wood, Andrea Pugh, Joanne Hammett, Rory Sheehan, Samuel Tromans, Rohit Shankar

**Affiliations:** Cornwall Partnership NHS Foundation Trust, Truro, UK; University of South Wales, Pontypridd, UK; Cornwall Intellectual Disability Equitable Research (CIDER), University of Plymouth Peninsula School of Medicine, Truro, UK; and Swansea Bay University Health Board, Port Talbot, UK; Statsconsultancy, Amersham, UK; Coventry and Warwickshire Partnership NHS Trust, Coventry, UK; Kent and Medway NHS and Social Care Partnership Trust, Gillingham, UK; Hertfordshire Partnership University NHS Foundation Trust, St Albans, UK; Central and Northwest London NHS Foundation Trust, London, UK; Swansea Bay University Health Board, Port Talbot, UK; Institute of Psychiatry, Psychology & Neuroscience, King's College London, London, UK; SAPPHIRE Group, Department of Population Health Sciences, University of Leicester, Leicester, UK; and Adult Learning Disability Service, Leicestershire Partnership NHS Trust, Leicester, UK; Cornwall Partnership NHS Foundation Trust, Truro, UK; and Cornwall Intellectual Disability Equitable Research (CIDER), University of Plymouth Peninsula School of Medicine, Truro, UK

**Keywords:** Seizures, intellectual disability, mental health, comorbidity, care setting

## Abstract

**Background:**

A quarter of People with Intellectual Disabilities (PwID) have epilepsy compared with 1% of the general population. Epilepsy in PwID is a bellwether for premature mortality, multimorbidity and polypharmacy. This group depends on their care provider to give relevant information for management, especially epilepsy. There is no research on care status relationship and clinical characteristics of PwID and epilepsy.

**Aim:**

Explore and compare the clinical characteristics of PwID with epilepsy across different care settings.

**Method:**

A retrospective multicentre cohort study across England and Wales collected information on seizure characteristics, intellectual disability severity, neurodevelopmental/biological/psychiatric comorbidities, medication including psychotropics/anti-seizure medication, and care status. Clinical characteristics were compared across different care settings, and those aged over and younger than 40 years.

**Results:**

Of 618 adult PwID across six centres (male:female = 61%:39%), 338 (55%) received professional care whereas 258 (42%) lived with family. Significant differences between the care groups existed in intellectual disability severity (*P* = 0.01), autism presence (*P <* 0.001), challenging behaviour (*P* < 0.001) and comorbid physical conditions (*P* = 0.008). The two groups did not vary in intellectual disability severity/genetic conditions/seizure type and frequency/psychiatric disorders. The professional care cohort experienced increased polypharmacy (*P* < 0.001) and antipsychotic/psychotropic use (*P* < 0.001/*P* = 0.008).

The over-40s cohort had lower autism spectrum disorder (ASD) and attention-deficit hyperactivity disorder (ADHD) comorbidity (*P* < 0.001/*P* = 0.007), increased psychiatric comorbidity and challenging behaviour (*P* < 0.05), physical multimorbidity (*P* < 0.001), polypharmacy (*P* < 0.001) and antipsychotic use (*P* < 0.001) but reduced numbers of seizures (*P* = 0.007).

**Conclusion:**

PwID and epilepsy over 40 years in professional care have more complex clinical characteristics, increased polypharmacy and antipsychotic prescribing but fewer seizures.

Intellectual disability is a neurodevelopmental condition characterised by global deficits in adaptive and cognitive functioning that begins in the developmental period.^1^ In the UK, 22% of people with intellectual disability (PwID) are estimated to have co-occurring epilepsy, compared with 1% in the general population.^2^ Prevalence of epilepsy increases with severity of intellectual disability.^2^ It is suggested that PwID have higher rates of epilepsy because of common or related causative mechanisms, which may include structural differences in brain development and genetic predisposition.^3^

PwID and epilepsy are underrepresented in the research literature.^4^ There is continued health inequity in PwID, with epilepsy recognised as the most common long-term health condition associated with premature mortality in PwID.^5,6^ An estimate from national mortality review programmes reveals that around 60% of PwID die before 65 years of age and that approximately one-third of those that die have epilepsy.^6^ The average number of long-term health conditions of the deaths reviewed was 2.48.^6^

PwID and epilepsy have increased rates of multimorbidity (two or more chronic conditions) and polypharmacy (five or more regular medicines), compared with the general population.^6–8^ Multimorbidity is closely associated with polypharmacy, which increases the risk of adverse drug reactions.^9^ A retrospective cohort study (*n* = 904) reviewing PwID and co-occurring epilepsy found that over half have a physical health comorbidity and a third have a psychiatric comorbidity.^10^ There is a three times higher likelihood of Sudden Unexpected Death in Epilepsy (SUDEP) in PwID than the general population.^11,12^

## Complex social support needs of PwID with epilepsy

Social care plays a significant role in epilepsy management.^13,14^ In this context, the term ‘social care’ broadly refers to non-medical, practical and personal support.^13,14^ PwID require significant social care, given the range of complex health and daily care needs.^13^ There is most likely a variation in the level of support that PwID with epilepsy receive in the community, dependent upon the complexity of their needs.^13,14^ It is recognised that care settings of PwID and epilepsy could influence outcomes, including issues such as the risk of SUDEP.^13–15^ It is also recognised that older PwID and epilepsy (for PwID defined as >40 years of age^16^) are more likely to be in professional care, having higher levels of multimorbidity and polypharmacy but are likely to receive fewer clinical reviews for their epilepsy.^16,17^ However, there is a limited evidence base regarding understanding the influence of social care provision on epilepsy outcomes and risk in PwID.^14^

This investigation explored the clinical characteristics of PwID with epilepsy across different care settings. It compares subgroups of those PwID who live independently or with family, with those who live with support of professional care staff.

## Method

An England and Wales multicentre retrospective cohort study was performed. The Strengthening the Reporting of Observational studies in Epidemiology (STROBE) checklist was utilised to design and report this study (Supplementary information 1, available at https://doi.org/10.1192/bjo.2024.45).

Data were collected from participating National Health Service (NHS) centres by case-note review of electronic health records. Participating sites were recruited through individual invitation in October 2022. The inclusion criteria were adults (aged 18 years and over) with a diagnosis of both intellectual disability and epilepsy, known to community learning disability and/or epilepsy health teams at the time of data collection. Data were entered onto a predesigned spreadsheet (Appendix 1). Information was collected on demographic status including gender and age, and the severity of intellectual disability divided into mild and moderate/profound; physical and psychiatric comorbidities; medication variables including number of medications, types of medication and as required (‘*pro re nata*' [p.r.n.]) medications; epilepsy data including seizure profile and presence of seizure in the 6 months prior to data collection. The living situation was defined as ‘professional’ (that is those living in state-sponsored care facilities), ‘living with family’ or ‘other’. Those living independently were categorised as other. No patient identifiable data were collected. Individual data-sets from each participating service were combined into a single data-set prior to analysis.

### Statistical analysis

The initial analysis was descriptive, summarising data for all study participants. An additional analysis further compared the ‘nature of care’ groups. Of particular interest was the people's residential status, ‘nature of care’ (i.e. whether living with family, with professional carer support or other setting), and the association between this factor and variables including polypharmacy. A subgroup analysis was completed for patients aged over 40 years, as age is considered a significant risk factor variable (older adult cohort).^16,17^ Comparisons were also made between those aged over and under 40.

All analyses were performed using regression methods. For all outcomes, an unadjusted analysis was performed, following by an analysis adjusted for age, gender and severity of intellectual disability. Binary outcomes were analysed using logistic regression, whereas ordinal logistic regression was used for ordinal outcomes. Multinomial logistic regression was used for categorical with unordered categories. One continuous outcome, number of medications, was analysed using linear regression. This was given a log transformation before analysis, due to its positively skewed distribution.

## Ethics statement

We confirm that we have read the journal's position on issues involved in ethical publication and affirm that this report is consistent with those guidelines.

### Ethics and governance

Each NHS centre registered the data collection as an audit/service evaluation and undertook Data Protection Impact Assessments (DIPA) in line with local policies. Approval was gained by each site from local information governance teams. Only de-identified data were submitted to the central data-set. This process was overseen by an information governance lead to ensure that data extraction and transfer was in compliance with the General Data Protection Regulation (GDPR). This study did not require formal ethical approval as per the NHS Health Research Authority tool (see http://www.hra-decisiontools.org.uk/research/index.html in Supplementary information 2). We confirm that we have read the journal's position on issues involved in ethical publication and affirm that this report is consistent with those guidelines.

### Data sharing

Anonymised participant data and the data dictionary are available along with the study protocol and can be requested from the corresponding author.

## Results

### Summary of study data for all participants (Supplementary information 3: Supplementary Tables 1–3)

Data were collected from 618 patients across six specialist centres in England and Wales. The mean age of the patient group was 39.9 years old (±14.5), and over half (61%) were male. The most common care setting was professional for 338 PwID (55%), whereas 258 PwID (42%) lived with their family. All analyses were compared between these two settings only. There were a small number of patients who received care in other settings (2.5%, *n* = 16) and a small number who did not report their care setting (*n* = 4). These were excluded from the analysis.

Three-quarters of patients (*n* = 460; 75%) had moderate/profound levels of intellectual disability severity. A fifth (*n* = 128, 21%) had one or more genetic conditions. Over a third (*n =* 234; 38%) of PwID were also autistic, and 4% (*n =* 22) had comorbid ADHD. A quarter of PwID had an affective disorder (*n =* 159; 26%), with a similar proportion having challenging behaviour (*n =* 160; 28%).

Having generalised seizures only was the most common type of seizure, which accounted for 59% of PwID (*n =* 361). Around two-thirds (65%; *n =* 400) of PwID had had a seizure in the previous 6 months.

A third (*n =* 212; 34%) of PwID had no diagnosed physical conditions, with 5% having five or more physical conditions (*n =* 29). Non-genetic epilepsy syndrome was present in 3% of PwID (*n =* 16).

Polypharmacy, defined as five or more medications, was present in over a third (*n =* 234; 38%) of patients. Patients had a median of five regular medications. A small number (*n =* 26; 4%) were prescribed more than ten medications.

Over 40% of patients had three or more anti-seizure medications (ASM) (*n =* 254; 41%), and 9% were on vagal nerve stimulation (VNS) therapy (*n =* 53). Over a quarter (*n =* 173; 28%) of PwID were on antipsychotics, whereas over two-thirds were on p.r.n. medications (*n =* 409; 68%). In the cohort as a whole, 17% of PwID experienced physical side effects to medications (*n =* 105), whereas 3% experienced psychiatric side effects (*n =* 21).

### Comparison between nature of care settings

#### Health conditions and seizure variables (Table 1)

Health conditions and seizure variables according to care setting are summarised in Table 1. There were a small number of PwID who received care in other settings (*n* = 16) and a small number whose care setting was not known/not reported (*n* = 4). These were excluded from the analysis. Of those in the analysis, 338 were in a professional setting and 260 were living with family.

**Table 1:** Health conditions and seizure variables by the nature of the care setting

Variable	Score	Professional		Living with family		Unadjusted		Adjusted^a^	
		*n*	*n* (%)	*n*	*n* (%)	Odds ratio (95% CI)^b^	*P*-value	Odds ratio (95% CI)^b^	*P*-value
Intellectual disability severity	Mild	338	72 (21)	258	70 (27)				
	Moderate/ Profound		266 (79)		188 (73)	0.73 (0.50, 1.06)	0.10	0.58 (0.38, 0.88)	**0**.**01**
Genetic conditions	No	338	270 (80)	260	200 (26)				
	Yes (1+)		68 (20)		60 (26)	1.19 (0.80, 1.76)	0.38	1.10 (0.72, 1.69)	0.66
Autism	No	338	196 (58)	260	176 (68)				
	Yes		142 (42)		84 (32)	0.66 (0.47, 0.92)	**0**.**02**	0.36 (0.24, 0.54)	**<0**.**001**
ADHD	No	338	325 (96)	260	252 (97)				
	Yes		13 (4)		8 (3)	0.79 (0.32, 1.94)	0.61	0.39 (0.15, 1.01)	0.05
Psychotic	No	338	303 (90)	260	246 (95)				
	Yes		35 (10)		14 (5)	0.49 (0.26, 0.94)	**0**.**03**	0.59 (0.29, 1.20)	0.15
Affective disorder	No	338	239 (71)	260	206 (79)				
	Yes		99 (29)		54 (21)	0.63 (0.43, 0.93)	**0**.**02**	0.66 (0.43, 1.00)	0.05
Challenging behaviour	No	312	196 (63)	239	199 (83)				
	Yes		116 (37)		40 (17)	0.34 (0.23, 0.51)	**<0**.**001**	0.34 (0.22, 0.53)	**<0**.**001**
Other psychiatric disorder	No	312	296 (95)	239	233 (97)				
	Yes		16 (5)		6 (3)	0.47 (0.18, 1.24)	0.13	0.81 (0.28, 2.34)	0.70
Seizure type	Generalised	335	196 (59)	259	157 (61)				
	Other		87 (26)		63 (24)				
	Both types		52 (16)		39 (15)	-	0.87	-	0.93
Seizures in last 6 months	No	335	125 (37)	257	75 (29)				
	Yes		210 (63)		182 (71)	1.44 (1.02, 2.05)	**0**.**04**	1.19 (0.81, 1.74)	0.38
Number of physical conditions	0	337	122 (36)	260	82 (32)				
	1		92 (27)		86 (33)				
	2–4		111 (33)		75 (29)				
	5+		12 (4)		17 (7)	1.12 (0.83, 1.50)	0.61	1.55 (1.12, 2.13)	**0**.**008**
Non-genetic	No	337	329 (98)	260	252 (97)				
epilepsy syndrome	Yes		8 (2)		8 (3)	1.31 (0.48, 3.53)	0.60	1.31 (0.45 3.79)	0.62

Significant results are indicated in bold, i.e. values of *p* < 0.05. ADHD, attention-deficit hyperactivity disorder.

a. Adjusted for age, gender and severity of intellectual disability.

b. Odds ratios expressed as the odds of the outcome for the group living with family relative to the odds for the group living in professional care.

There are statistically significant differences between the nature of the two care groups in the presence of autism (*P =* 0.02), psychotic illness (*P =* 0.03), affective disorders (*P =* 0.02) and reported frequency of seizures in the previous 6 months (*P =* 0.04). However, the two groups did not vary in intellectual disability severity, presence of genetic conditions, ADHD, other psychiatric disorders, seizure type, number of physical conditions or the presence of non-genetic epilepsy syndrome.

Autism was significantly more common in those in professional settings. A total of 42% of PwID in this setting were autistic (*n =* 142), compared with 32% of PwID living with their family (*n =* 84). Psychotic illness was also more common in the professional setting group (10%; *n =* 35), compared with 5% of PwID living with family (*n =* 14). Affective disorders (*P =* 0.02) and challenging behaviour (*P* < 0.001) were both significantly more common in the group living in a professional setting. Over a third (37%; *n* = 116) of PwID in a professional setting had challenging behaviour, compared with only 17% (*n =* 40) of those living with family.

Seizures in the previous 6 months were slightly less common in PwID in professional settings where 63% (*n =* 210) in this setting had seizures in this time period, which contrasted with 71% (*n =* 182) of those living with family.

When the comparison of health conditions was adjusted for age, gender and severity of intellectual disability, further significant differences were identified. People with moderate/profound intellectual disability were significantly more likely to be living in a professional care setting (odds ratio, 95% CI: 0.58 [0.38, 0.88], *P* = 0.01). The group living with family were significantly more likely to have comorbid physical health conditions (1.55 [1.12, 2.13], *P* = 0.008). Initial significant findings of a difference in risk of psychotic illness, affective disorders and seizure frequency were no longer significant following adjustment.

#### Medication and medication side effects

The two groups were also compared in terms of medication variables (Table 2). Statistically significant differences for the total number of medications given (*P* < 0.001) was found between the nature of the two care groups. This was the case when the number was considered as a continuous variable, in four categories or by defining polypharmacy. The professional setting group had more medications in total. The median number was five medications for the group living in professional settings and four for the group living with family. Almost half (48%; *n =* 161) had polypharmacy in the professional setting group, compared with 26% (*n =* 67) in the group living with family.

**Table 2:** Medications and medication side effects by the nature of the care setting

Variable	Category	Professional		Living with family		Unadjusted		Adjusted^a^	
		*n*	Summary	*n*	Summary	Odds ratio (95% CI)^b^	*P*-value	Odds ratio (95% CI)^b^	*P*-value
Total number medications^c^	−	338	5 [4, 7]	258	4 [3, 6]	0.82 (0.77, 0.87)	**<0.001**	0.88 (0.82, 0.95)	**<0**.**001**
Total number medications (categorised)	≤2	338	21 (6%)	260	48 (18%)				
	3–5		270 (46%)		200 (56%)				
	6–10		68 (42%)		60 (23%)				
	11+		20 (6%)		5 (2%)	0.35 (0.26, 0.49)	**<0**.**001**	0.48 (0.34, 0.68)	**<0**.**001**
Polypharmacy (5+ medications)	No	338	177 (52%)	260	193 (74%)				
	Yes		161 (48%)		67 (26%)	0.38 (0.27, 0.54)	**<0**.**001**	0.52 (0.35, 0.75)	**0**.**001**
ASM	0	338	4 (1%)	260	3 (1%)				
	1–2		194 (57%)		148 (57%)				
	3–4		123 (36%)		94 (36%)				
	5+		17 (5%)		15 (6%)	1.03 (0.75, 1.42)	0.85	1.04 (0.73, 1.47)	0.84
VNS	No	338	313 (93%)	259	232 (90%)				
	Yes		25 (7%)		27 (10%)	1.46 (0.82, 2.58)	0.20	1.10 (0.60, 2.01)	0.77
Antipsychotics	0	338	216 (64%)	239	214 (82%)				
	1		114 (34%)		39 (15%)				
	2+		8 (2%)		7 (3%)	0.39 (0.27, 0.58)	**<0**.**001**	0.46 (0.30, 0.69)	**<0**.**001**
Other psychotropic medications	0	337	213 (63%)	260	192 (74%)				
	1		106 (31%)		61 (23%)				
	2+		18 (5%)		7 (3%)	0.60 (0.42, 0.86)	**0**.**02**	0.60 (0.41, 0.88)	**0**.**008**
p.r.n. medications	0	332	96 (29%)	255	89 (35%)				
	1–2		192 (58%)		149 (58%)				
	3+		44 (13%)		17 (7%)	0.68 (0.49, 0.94)	**0**.**02**	0.68 (0.48, 0.96)	**0**.**03**
Physical side effects	No	338	272 (80%)	260	227 (87%)				
	Yes		66 (20%)		33 (13%)	0.60 (0.38, 0.94)	**0**.**03**	0.69 (0.42, 1.12)	0.13
Psychiatric side effects	No	338	326 (96%)	260	252 (97%)				
	Yes		12 (4%)		8 (3%)	0.86 (0.35, 2.14)	0.75	1.02 (0.38, 2.71)	0.97

Significant results are indicated in bold, i.e. values of *p* < 0.05.

Summary statistics: number (percentage) or median [inter-quartile range]. ASM, anti-seizure medication; VNS, vagal nerve stimulation; p.r.n., as needed ('*pro re nata*').

a. Adjusted for age, gender and severity of intellectual disability.

b. Odds ratios expressed as the odds of the outcome for group living with family relative to the odds for the group living in professional care.

c. Group difference expressed as the ratio of the number of medications for those living with family relative to the number for those in professional care.

Antipsychotics, other psychotropic medication and p.r.n. medication were also significantly more common in the professional setting group (*P <* 0.001). Over a third (36%; *n =* 122) of the professional setting group received antipsychotics, compared with only 18% (*n =* 46) of the group living with family. There were no differences for ASM and VNS use between the two groups.

Physical side effects of medication were significantly more common in the professional setting group (*P =* 0.03), occurring in 20% (*n =* 66) of PwID, compared with 13% (*n =* 33) of those living with family. The groups did not significantly vary in terms of psychiatric side effects.

When the comparison of medication and medication side effects was adjusted for age, gender and severity of intellectual disability, there was no longer a significant difference in the presence of physical side effects. There was no significant difference to the unadjusted data for any other parameter measured.

### Comparison between age categories (age ≤ 40 and age > 40)

#### Health and seizure variables (Table 3)

The age groups were compared in terms of health and seizure variables (Table 3). There were 358 PwID who were aged 40 or younger, whereas 260 PwID were aged over 40.

**Table 3:** Health conditions and seizure variables by age group

Variable	Score	Age ≤ 40		Age > 40		Unadjusted		Adjusted^a^	
		*n*	*n* (%)	*n*	*n* (%)	Odds ratio (95% CI)^b^	*P*-value	Odds ratio (95% CI)^b^	*P*-value
Intellectual disability severity	Mild	357	82 (23)	259	74 (29)				
	Moderate/ profound		275 (77)		185 (71)	0.75 (0.52, 1.07)	0.11	0.75 (0.52, 1.08)	0.13
Genetic conditions	No	358	281 (78)	260	209 (80)				
	Yes (1+)		77 (22)		51 (20)	0.89 (0.60, 1.32)	0.57	0.92 (0.61, 1.37)	0.67
ASD	No	357	189 (53)	260	194 (75)				
	Yes		168 (47)		66 (25)	0.38 (0.27, 0.54)	**<0**.**001**	0.40 (0.28, 0.57)	**<0**.**001**
ADHD	No	357	336 (96)	260	259 (99.6)				
	Yes		21 (6)		8 (0.4)	0.06 (0.01, 0.46)	**0**.**007**	0.06 (0.01, 0.46)	**0**.**007**
Psychotic disorder	No	357	338 (95)	260	246 (88)				
	Yes		19 (5)		31 (12)	2.41 (0.33, 4.37)	**0**.**004**	2.23 (1.22, 4.09)	**0**.**009**
Affective disorder	No	357	285 (80)	260	173 (67)				
	Yes		72 (20)		87 (33)	1.99 (1.38, 2.87)	**<0**.**001**	1.91 (1.32, 2.77)	**0**.**001**
Challenging behaviour	No	330	246 (75)	236	160 (68)				
	Yes		84 (25)		76 (32)	1.39 (0.96, 2.01)	0.08	1.56 (1.06, 2.28)	**0**.**02**
Other psychiatric disorder	No	330	324 (98)	236	220 (93)				
	Yes		6 (2)		16 (7)	3.93 (1.51, 10.2)	**0**.**005**	3.66 (1.40, 9.56)	**0**.**008**
Seizure type	Generalised	335	221 (62)	257	140 (54)				
	Other		81 (23)		78 (30)				
	Both types		54 (15)		39 (15)	−	0.09	−	0.16
Seizures in last 6 months	No	352	105 (30)	259	106 (41)				
	Yes		247 (70)		153 (59)	0.61 (0.44, 0.86)	**0**.**004**	0.63 (0.45, 0.88)	**0**.**007**
Number of physical conditions	0	357	146 (41)	257	66 (25)				
	1		110 (31)		78 (30)				
	2–4		87 (24)		100 (39)				
	5+		14 (4)		15 (6)	1.99 (1.48, 2.67)	**<0**.**001**	2.01 (1.50, 2.71)	**<0**.**001**
Non-genetic	No	358	347 (97)	259	254 (98)				
epilepsy syndrome	Yes		11 (3)		5 (2)	0.62 (0.21, 1.81)	0.38	0.70 (0.24, 2.07)	0.52

Significant results are indicated in bold, i.e. values of *p* < 0.05. ASD, autism spectrum disorder; ADHD, attention-deficit hyperactivity disorder.

a. Adjusted for gender and severity of intellectual disability.

b. Odds ratios expressed as the odds of the outcome for the group aged >40 relative to the odds for the group aged ≤40.

Autism (*P <* 0.001) and ADHD (*P <* 0.001) were significantly more common in the younger age group. Almost half (*n* = 168, 47%) of those aged 40 and younger were autistic, compared with a quarter (*n* = 66, 25%) of PwID aged over 40. Conversely, psychotic illness, affective disorders and other psychiatric disorders were less common in the younger age group, compared with older patients. One in five of the younger age group had an affective disorder, which contrasted with one-third of the older age group.

The two groups were not significantly different for level of intellectual disability severity, the presence of genetic conditions and non-genetic epilepsy syndrome. There was some evidence of a difference for challenging behaviour, but this difference did not reach statistical significance.

The seizure variables suggested no evidence of a statistically significant group difference for seizure type (*P =* 0.09). However, seizures in the previous 6 months were found to be significantly different between groups (*P =* 0.004). Recent seizures were more common in the younger group, with 247 (70%) having seizures in the previous 6 months, compared with 153 (59%) of the older age group.

The older age group had a significantly higher number of physical conditions than the younger group (*P <* 0.001). Almost half (*n* = 115, 45%) of the over 40s had multimorbidity as defined as two or more conditions, compared with just over a quarter (*n* = 101, 28%) of the younger group.

When the health conditions and seizure variables were adjusted for gender and severity of intellectual disability, the risk of challenging behaviour was significantly more likely in the older adult group (1.56 (1.06, 2.28) *P* = 0.02).

#### Medication and medication side effects (Table 4)

The two age groups were also compared in terms of the medication variables. Analysis results are summarised in Table 4. The analyses suggested statistically significant differences between age groups for the total number of medications (*P <* 0.001), use of VNS (*P =* 0.007) and antipsychotic (*P =* 0.002) medication. There was no evidence of a statistically significant difference between age groups for psychotropic medication. The two groups did not differ significantly in terms of ASM and p.r.n. medication.

**Table 4:** Medications and medication side effects by age group

Variable	Category	Age ≤ 40		Age > 40		Unadjusted		Adjusted^a^	
		*n*	Summary	*n*	Summary	Odds ratio (95% CI)^b^	*P*-value	Odds ratio (95% CI)^b^	*P*-value
Total number medications ^c^	−	357	4 [3, 6]	260	6 [4, 8]	1.29 (1.21, 1.38)	**<0.001**	1.29 (1.21, 1.37)	**<0**.**001**
Total number medications (categorised)	≤2	357	62 (6%)	260	13 (5%)				
	3–5		202 (57%)		106 (41%)				
	6–10		83 (23%)		125 (48%)				
	11+		10 (3%)		16 (6%)	3.38 (2.46, 4.65)	**<0**.**001**	3.42 (2.47, 4.72)	**<0**.**001**
Polypharmacy (5+ medications)	No	357	264 (74%)	260	119 (46%)				
	Yes		93 (26%)		141 (54%)	3.36 (2.40, 4.72)	**<0**.**001**	3.32 (2.36, 4.68)	**<0**.**001**
ASM	0	356	4 (1%)	260	4 (2%)				
	1–2		208 (58%)		146 (56%)				
	3–4		123 (35%)		99 (38%)				
	5+		21 (6%)		11 (4%)	1.03 (0.75, 1.42)	0.85	1.04 (0.76, 1.43)	0.81
VNS	No	357	317 (89%)	259	246 (95%)				
	Yes		40 (11%)		13 (5%)	0.42 (0.22, 0.80)	**0**.**008**	0.42 (0.22, 0.81)	**0**.**01**
Antipsychotics	0	357	276 (77%)	239	168 (65%)				
	1		74 (21%)		84 (32%)				
	2+		7 (2%)		8 (3%)	1.86 (1.30, 2.64)	**0**.**001**	1.91 (1.33, 2.73)	**<0**.**001**
Other psychotropic medications	0	357	254 (71%)	259	163 (63%)				
	1		87 (24%)		85 (33%)				
	2+		16 (4%)		11 (4%)	1.41 (1.01, 1.98)	0.07	1.37 (0.98, 1.93)	0.07
p.r.n. medications	0	352	113 (32%)	253	83 (33%)				
	1–2		205 (58%)		142 (56%)				
	3+		34 (10%)		28 (11%)	1.01 (0.74, 1.39)	0.94	1.07 (0.77, 1.48)	0.68
Physical side effects	No	357	299 (84%)	260	213 (82%)				
	Yes		58 (16%)		47 (18%)	1.14 (0.75, 1.74)	0.55	1.19 (0.78, 1.82)	0.43
Psychiatric side effects	No	357	346 (97%)	260	250 (96%)				
	Yes		11 (3%)		10 (4%)	1.26 (0.53, 3.01)	0.61	1.35 (0.56, 3.26)	0.50

Significant results are indicated in bold, i.e. values of *p* < 0.05. Summary statistics: number (percentage) or median [inter-quartile range]. ASM, anti-seizure medication; VNS, vagal nerve stimulation; p.r.n., as needed ('*pro re nata*').

a. Adjusted for gender and severity of intellectual disability.

b. Odds ratios expressed as the odds of the outcome for the group aged >40 relative to the odds for the group aged ≤40.

c. Group difference expressed as the ratio of the number of medications for the group living with family relative to the number for those in professional care.

Pwintellectual disability aged 40 or under had a median of four medications, which contrasted with a median of six for those aged over 40. Over half (*n* = 141, 54%) of older patients had polypharmacy, compared with only a quarter (*n* = 93, 26%) of the younger age group. Antipsychotic medication was also more common in the older age group, with 92 (35%) on this type of medication, compared with 81 (23%) of the younger age group. Conversely, VNS use was more common in the younger age group (*n* = 40, 11%), compared with only (*n* = 13, 5%) of the older age group. Neither the presence of physical or psychiatric side effects demonstrated statistically significant differences between the two age groups.

There were no significant differences in comparison with the unadjusted results when data were adjusted for gender and severity of intellectual disability.

### Over-40s comparison based on nature of care setting

Of 260 people aged over 40, the nature of care for three PwID was not known/not reported, and a further six individuals had an ‘other’ care setting. These nine people were omitted from the analysis, leaving 251 for analysis. Within this group, 190 (76%) had a professional care setting, whereas 61 (24%) lived with family. Health condition and seizure variables for the nature of care groups are reported in Table 5.

**Table 5:** Health conditions and seizure variables by the nature of the care setting (age >40 years)

Variable	Score	Professional		Living with family		Unadjusted		Adjusted^a^	
		*n*	*n* (%)	*n*	*n* (%)	OR (95% CI)^b^	*P*-value	OR (95% CI)^b^	*P*-value
Intellectual disability severity	Mild	190	52 (27)	60	17 (28)				
	Moderate/ Profound		138 (73)		43 (72)	0.95 (0.50, 1.82)	0.88	0.93 (0.48, 1.81)	0.83
Genetic conditions	No	190	151 (79)	61	49 (80)				
	Yes (1+)		39 (21)		12 (20)	0.95 (0.46, 1.95)	0.89	0.84 (0.40, 1.78)	0.65
ASD	No	190	134 (71)	61	55 (90)				
	Yes		56 (29)		6 (10)	0.26 (0.11, 0.64)	**0**.**003**	0.17 (0.06, 0.44)	**<0**.**001**
ADHD	No	190	189 (99)	61	61 (100)				
	Yes		1 (1)		0 (0)	^c^	1.00	−	−
Psychotic disorder	No	190	165 (87)	61	56 (92)				
	Yes		25 (13)		5 (8)	0.59 (0.22, 1.61)	0.30	0.72 (0.25, 2.08)	0.54
Affective disorder	No	190	125 (66)	61	43 (70)				
	Yes		65 (34)		18 (30)	0.81 (0.43, 1.51)	0.50	0.78 (0.41, 1.49)	0.45
Challenging behaviour	No	174	108 (62)	56	47 (84)				
	Yes		66 (38)		9 (16)	0.31 (0.14, 0.68)	**0**.**003**	0.29 (0.13, 0.64)	**0**.**002**
Other psychiatric disorder	No	174	161 (93)	56	53 (95)				
	Yes		13 (7)		3 (5)	0.70 (0.19, 2.55)	0.59	0.92 (0.23, 3.62)	0.91
Seizure type	Generalised	188	99 (53)	60	36 (60)				
	Other		59 (31)		16 (27)				
	Both types		30 (16)		8 (13)	−	0.61	−	0.27
Seizures in last 6 months	No	189	76 (40)	61	26 (43)				
	Yes		113 (60)		35 (57)	0.91 (0.50, 1.62)	0.74	0.80 (0.43, 1.46)	0.46
Number of physical conditions	0	189	53 (28)	61	10 (16)				
	1		54 (29)		19 (31)				
	2–4		72 (38)		27 (44)				
	5+		10 (5)		5 (8)	1.59 (0.93, 2.69)	0.09	1.84 (1.06, 3.21)	**0**.**03**
Non-genetic epilepsy syndrome	No	189	285 (98)	61	60 (98)				
	Yes		4 (2)		1 (2)	0.77 (0.08, 7.03)	0.82	0.58 (0.06, 5.48)	0.64

Significant results are indicated in bold, i.e. values of *p* < 0.05. ASD, autism spectrum disorder; ADHD, attention-deficit hyperactivity disorder.

a. Adjusted for age, gender and severity of intellectual disability.

b. Odds ratios expressed as the odds of the outcome for the group living with family relative to the odds for the group living in professional care.

c. Unable to calculate the odds ratios due to no occurrences in one group. Analysis using Fisher's exact test.

Statistically significant differences between the nature of the two care groups are found for the presence of autism (*P =* 0.002) and challenging behaviour (*P =* 0.002). However, the two groups did not demonstrate statistically significant differences for the other variables examined.

Autism and challenging behaviour were more common in PwID living in professional settings with 29% (*n =* 56) of PwID reported autistic, compared with 10% (*n =* 6) of those living with family. Challenging behaviour occurred in over a third (*n =* 66, 38%) of patients in a professional setting, compared with 16% (*n =* 9) of those living with family.

Medication variables are summarised in Table 6. Statistically significant differences were found between the nature of the two care groups for the total number of medications given (*P =* 0.02). This was the case when the number was considered as a continuous variable, in four categories or by defining polypharmacy. The professional group had more medications in total. The median number was six medications for the group living in professional settings, but there was a median of five for the group living with family. Over half (58%; *n =* 111) in the professional setting group had polypharmacy , compared with 41% (*n =* 25) in the group living with family. Antipsychotics were significantly more common in the professional setting group (*P =* 0.03) with 40% (*n =* 76) of them receiving antipsychotics, compared with 21% (*n =* 13) of the group living with family.

**Table 6:** Medications and medication side effects by the nature of the care setting (age >40 years)

Variable	Category	Professional		Living with family		Unadjusted		Adjusted^a^	
		*n*	Summary	*n*	Summary	OR (95% CI)^b^	*P*-value	OR (95% CI)^b^	*P*-value
Total number medications^c^	−	190	6 [5,8]	61	5 [4,7]	0.88 (0.79, 0.97)	**0.02**	0.90 (0.81, 1.00)	0.06
Total number medications (categorised)	≤2	190	6 (3%)	61	5 (8%)				
	3–5		73 (38%)		31 (51%)				
	6–10		97 (51%)		24 (39%)				
	11+		14 (7%)		1 (2%)	0.46 (0.26, 0.80)	**0.006**	0.51 (0.28, 0.90)	**0**.**02**
Polypharmacy (5 + medications)	No	190	79 (42%)	61	36 (59%)				
	Yes		111 (58%)		25 (41%)	0.49 (0.28, 0.89)	**0.02**	0.54 (0.29, 0.99)	**0**.**04**
ASM	0	190	2 (1%)	61	1 (2%)				
	1–2		108 (57%)		34 (56%)				
	3–4		73 (38%)		22 (36%)				
	5+		7 (4%)		4 (7%)	1.06 (0.59, 1.88)	0.85	1.03 (0.57, 1.86)	0.93
VNS	No	190	181 (95%)	60	56 (93%)				
	Yes		9 (5%)		4 (7%)	1.44 (0.43, 4.84)	0.56	1.29 (0.37, 4.55)	0.69
Antipsychotics	0	190	114 (60%)	61	48 (79%)				
	1		69 (36%)		12 (20%)				
	2+		7 (4%)		1 (2%)	0.41 (0.21, 0.80)	**0**.**009**	0.43 (0.21, 0.85)	**0**.**02**
Other psychotropic medications	0	189	114 (60%)	61	43 (70%)				
	1		66 (35%)		17 (28%)				
	2+		9 (5%)		1 (2%)	0.62 (0.34, 1.15)	0.13	0.64 (0.33, 1.21)	0.17
p.r.n. medications	0	185	60 (32%)	60	19 (32%)				
	1–2		101 (55%)		37 (62%)				
	3+		24 (13%)		4 (7%)	0.88 (0.50, 1.53)	0.64	0.87 (0.48, 1.57)	0.65
Physical side effects	No	190	156 (82%)	61	50 (82%)				
	Yes		34 (18%)		31 (18%)	1.01 (0.48, 2.14)	0.98	1.17 (0.53, 2.56)	0.69
Psychiatric side effects	No	190	181 (95%)	61	60 (98%)				
	Yes		9 (5%)		1 (2%)	0.34 (0.04, 2.71)	0.31	0.33 (0.04, 2.76)	0.31

Significant results are indicated in bold, i.e. values of *p* < 0.05. Summary statistics: number (percentage) or median [inter-quartile range]. ASM, anti-seizure medication; VNS, vagal nerve stimulation; p.r.n., as needed ('*pro re nata*').

a. Adjusted for age, gender and severity of intellectual disability.

b. Odds ratios expressed as the odds of the outcome for the group living with family relative to the odds for the group living in professional care.

c. Group difference expressed as the ratio of the number of medications for those living with family relative to the number for those in professional care.

There were no statistically significant differences between the two groups for ASM, VNS, other psychotropic and p.r.n. medications. The occurrence of neither physical nor psychiatric side effects of medication varied significantly between people living in professional settings, and those living with family.

When adjusted for age, gender and severity of intellectual disability, there was no longer a significant difference in total number of medications prescribed between settings in the over 40 group. The adjustment did identify a significantly higher risk of physical health conditions in the group living with family (1.84 (1.06, 3.21) *P* = 0.03).

## Discussion

As the UK strives to provide greater opportunity and choice of care setting for PwID, this study provides clinically useful information to help understand the differences in clinical characteristics present across different care settings.

Three-quarters of the PwID are diagnosed with moderate/profound intellectual disability, illustrating the positive correlation between level of intellectual disability and seizures.^2^ In this study, 38% were diagnosed with ASD and 4% diagnosed with ADHD. The levels of comorbid neurodevelopmental disorders in this sample are similar to those in previous research in the PwID and epilepsy populations.^10^

In this study, 55% were found to live in professional care settings and 42% with family. National data for England record that 78.3% of adults with intellectual disability live in community, either with family or in their own homes, and 21.7% live in residential, nursing or hospital settings.^18^ The higher proportion living in residential care in this study is likely contributed to by the complexities of needs for this patient group with the dual diagnosis of intellectual disability and epilepsy.

Polypharmacy is present in over half of this sample. Antipsychotics were prescribed to 28%, although only 8% had a diagnosed psychotic disorder. This is similar to findings of previous studies,^10^ and further demonstrates that in PwID and epilepsy populations, there are high levels of antipsychotic prescribing for non-psychotic matters. This is of particular interest in this group as antipsychotic medications have the potential to lower the seizure threshold, and therefore it is imperative to ensure that prescription of antipsychotics is clinically justified and closely monitored. A significant proportion of antipsychotics prescribed for other reasons may be targeted at behaviours perceived as challenging, present in 28% of the total cohort. Behaviours that challenge can be difficult to clinically differentiate from seizure presentations, and this difficulty could contribute to high levels of both antipsychotic and ASM medications.^19^

Interestingly, behaviour perceived as challenging was more than twice as likely to be present in those living in professional care (37%), compared with those in a family environment (17%). This may be linked to the finding that professional care is associated with significantly increased rates of neurodevelopmental and psychiatric comorbidities (autism, affective disorders and psychosis).

Professional care settings were associated with an increased number of overall medications, presence of polypharmacy, antipsychotic prescribing, other psychotropic medication and p.r.n. medications. The group in professional care settings had a median of five medications, where for those living with family the median number of medications was four. There was no difference in the number of physical health comorbidities between the two groups. Twice as many in professional care settings receive antipsychotics compared with those living with family (36% versus 18%). It is possible that this may reflect an increased likelihood of complexity in physical and mental health comorbidity in those living in professional care. It is also possible that this reflects differences in ways of supporting those with challenging behaviours in professional versus family environments. It should be noted that physical side effects of medications were more common in those living in professional care settings. It could also be linked to a lack of health-specific knowledge and training, poor resources to engage PwID socially and less appreciation of the complexity of holistic care.^13^

### Subgroup analysis: PwID with epilepsy aged 40 years and over

PwID aged 40 years and over were more likely to live in professional care than the overall cohort (76% versus 55%). This may reflect increased care needs as people age, and/or the lack of ability of family members (often parents) to continue to meet the people's care needs.

The older adult cohort were prescribed a greater number of medications and had increased rates of polypharmacy and antipsychotic prescribing. PwID aged under 40 years had a median of four medications, with a median of six for those aged 40 and over. Furthermore, those in the older adult group living in professional care settings were on a median of six medications compared with five for those living with family. In the older adult cohort, those people living in professional care settings were more likely to be prescribed antipsychotics. This suggests an increased risk of polypharmacy in professional settings for those aged over 40.

There is evidence to suggest a strong association between PwID and epilepsy in those aged over 40, and a risk of polypharmacy.^16^ This study highlights that there is also an association with increased risk for those of any age living in a professional care setting, compared with those living with family. The use of antipsychotics is also higher for older adults in professional care settings. These findings have been replicated in previous studies. A population study in Sweden identified increased antipsychotic prescribing for PwID who live in supported housing (professional care) compared with those in community,^20^ and place of care has previously been associated with increased polypharmacy in older PwID, independent of other factors including severity of intellectual disability.^21^ Our study is the first to associate the subgroup of PwID and epilepsy in care to be of higher risk of polypharmacy in an already vulnerable population. There is pressing need not only to identify this problem but to reconcile it.^22,23^

The significant finding of recent seizures being more common in the younger group (70%) than older PwID (59%) in the previous 6 months is an interesting one. A recent review found that the likelihood of prevalence of seizure freedom in PwID for at least 1 year increased with age.^24^ Conversely, there is evidence to suggest that multiple ASM prescribing goes up for PwID with each decade of life.^24^ In addition, multiple ASM prescribing is linked also with a higher chance of other psychotropics prescribing including antipsychotics.^24^

Seen together, these patterns suggest the possibility of the use of ASMs for management for behavioural concerns particularly in older PwID.^25^

Other neurodevelopmental disorders (ASD and ADHD) were more frequently diagnosed in the younger age group. ASD was diagnosed twice as often in the younger cohort than in those aged 40 and over. This is reflected in previous literature demonstrating a diagnostic gap in adults aged over 50 years compared with younger people.^26^ Neurodevelopmental disorders were not routinely diagnosed until the end of the 20th century,^27^ with many of those older adults with epilepsy and intellectual disability therefore being part of a ‘underdiagnosed, lost generation’ of neurodevelopmental disorders.

### Mediation analysis discussion

The data suggest some evidence (although not strong) of an association between intellectual disability severity and nature of care (*P* = 0.10), a difference which is stronger when age/gender is considered (Table 1). Thus, this result potentially suggests some mediation effect of intellectual disability severity on the relationship between nature of care and the outcomes. There is only a relatively weak relationship between intellectual disability severity and age, which is the second ‘predictor’ variable (Table 3). This suggests there is little mediating effect of intellectual disability severity on the relationships between age group and outcomes.

### Limitations

This study used routinely collected data from health records and therefore has numerous limitations. It is able only to identify correlation and patterns. The study is retrospective and involved review of clinical records not specifically designed for the purpose of data collection. There are also likely differences in clinical settings and professional practices between services contributing to the study. The cohort studied is complex and derived from specialist services. Therefore, any results must be interpreted in the context of a restricted population.

### Implications for clinical practice

This study is the first to highlight differing clinical profiles of PwID and epilepsy dependent on the nature of care setting (family compared with professional) in a UK population. The study suggests that those aged 40 years and over living in professional care settings may have the most complex clinical characteristics. It identifies that both age (older adults >40) and care setting correlate significantly with increased number of medications and use of antipsychotics. These clinical factors have been associated with mortality risk. Additionally, considering their increased mortality risk, the NHS ‘Stopping Over-Medication of People’ with a learning disability, autism or both (STOMP) programme, focused on ‘helping people to stay well and have a good quality of life’,^28^ should consider particularly focusing on these patient groups. Prescribing generally in older PwID is a challenging issue, particularly if they have epilepsy.^29,30^ Neurologists and psychiatrists need to be aware of these concerns while managing care in this vulnerable population to prevent iatrogenic harm and to ensure that offered management is in the best interest of the individual. Reducing harm in this complex population would be a step towards closing the gap in health inequalities and premature mortality experienced by PwID and epilepsy.

### Implications for research

A larger-scale study exploring the development of clinical risk assessment decision tools may lead to better risk identification and mitigation. There is a need to reconsider how we approach understanding complex clinical populations and their care needs through research. The results from this study highlight some differences in clinical characteristics of the population that may be related to level of risk. However, there is a need for further understanding of the level of care provision provided, particularly by families, and how risk is managed. The data particularly highlight the increased physical health needs of those PwID living with families. An in-depth review would consider access to care, specialist support and any barriers faced. Furthermore, this review is based upon a cohort of people known to services and accessing care. This will not be representative of all PwID. Those people not known to services may be the hardest to reach and engage in research, and the most vulnerable. This includes PwID who are without a fixed home. This study does not focus on ethnicity; however, we know that people from ethnic minority communities may find it hardest to access services, and specific approaches are required to support participation in research.^31^

### Implications for training and policy

It might be that bespoke training focused on professional care providers needs considering, enumerating the specific concerns identified. Training care professionals on understanding health complexity and on identifying possible ‘domino effects’ of negative influences of multimorbidity and polypharmacy might assist in better outcomes. It could be a part of ongoing basic epilepsy training.^32^ This might require specific informed policy from good practice guidance and stakeholder organisations as has been achieved for SUDEP communication.^33,34^ The objective needs to be to develop ‘capable communities’ to support this vulnerable and complex group across different setting and across the age span.^35^

## Supporting information

Badger et al. supplementary material 1 Badger et al. supplementary material

Badger et al. supplementary material 2 Badger et al. supplementary material

## Data Availability

The data that support the findings of this study are available from the corresponding author upon reasonable request.

## Appendix 1 Data collection tool

**Table:**

Domain	Subject of interest	Subtype
	Gender	
Health	Intellectual disability severity	Mild or moderate/profound
	Genetic condition	
	Physical condition	
	Neurodevelopmental condition	ASD or ADHD
	Psychiatric diagnosis	Psychotic, affective, challenging behaviours, other
Seizure profile	Seizure type	
	Seizure in last 12 months	
Medication	Number of regular medications	
	Anti-seizure medication	
	Vagal nerve stimulator	
	Antipsychotics	
	Other psychotropic medication	
	p.r.n. medication	
	Side effects	Physical or psychiatric
Nature of care	Professional or living with family or other	
