# Supplementary material for: The relationship between clinical presentation and the nature of care in adults with intellectual disability and epilepsy – national comparative cohort study
Source: BJPsych Open. 2024 Apr 30;10(3):e94. doi: 10.1192/bjo.2024.45 (PMC11060072; doi:10.1192/bjo.2024.45)
Supplement: Badger et al. supplementary material 2 — Badger et al. supplementary material [file S2056472424000450sup002.docx]

*Supplementary information table 1: Demographic details of study participants*

| Variable | n | Category | Summary |
| --- | --- | --- | --- |
| Age | 618 | - | 39.9 ± 14.5 [18, 81] |
|  |  |  |  |
| Gender | 618 | Male | 376 (61%) |
|  |  | Female | 242 (39%) |
|  |  |  |  |
| Nature of care | 614 | Professional | 338 (55%) |
|  |  | Live with family | 260 (42%) |
|  |  | Other | 16 (3%) |

Summary statistics are: number (percentage) or mean ± standard deviation [range]

*Supplementary information table 2: Health conditions and seizure variables*

| Variable | n | Category | Number (%) |
| --- | --- | --- | --- |
|  |  |  |  |
| ID severity | 616 | Mild | 156 (25%) |
|  |  | Moderate – Profound | 460 (75%) |
|  |  |  |  |
| Number of genetic conditions | 618 | 0 | 490 (79%) |
|  |  | 1 | 126 (20%) |
|  |  | 2 | 1 (0.2%) |
|  |  | 3 | 1 (0.2%) |
|  |  |  |  |
| Autism Spectrum Disorder (ASD) | 617 | No | 383 (62%) |
|  |  | Yes | 234 (38%) |
|  |  |  |  |
| Attention deficit hyperactive disorder (ADHD) | 617 | No | 595 (96%) |
|  |  | Yes | 22 (4%) |
|  |  |  |  |
| Psychotic disorder | 617 | No | 567 (92%) |
|  |  | Yes | 50 (8%) |
|  |  |  |  |
| Affective disorder | 617 | No | 458 (74%) |
|  |  | Yes | 159 (26%) |
|  |  |  |  |
| Challenging behaviour | 566 | No | 406 (72%) |
|  |  | Yes | 160 (28%) |
|  |  |  |  |
| Other psychiatric disorder | 566 | No | 544 (96%) |
|  |  | Yes | 22 (4%) |
|  |  |  |  |
| Seizure type | 613 | Generalised | 361 (59%) |
|  |  | Other | 159 (26%) |
|  |  | Both types | 93 (15%) |
|  |  |  |  |
| Seizures in last 6 months | 611 | No | 211 (35%) |
|  |  | Yes | 400 (65%) |
|  |  |  |  |
| Number of diagnosed physical conditions | 616 | 0 | 212 (34%) |
|  |  | 1 | 188 (31%) |
|  |  | 2 – 4 | 187 (30%) |
|  |  | 5+ | 29 (5%) |
|  |  |  |  |
| Non-genetic epilepsy syndrome | 617 | No | 601 (97%) |
|  |  | Yes | 16 (3%) |
|  |  |  |  |

*Supplementary information table 3: Medications and medication side effects*

| Variable | n | Category | Summary |
| --- | --- | --- | --- |
|  |  |  |  |
| Total number regular medications | 617 | - | 5 [3, 7] |
|  |  |  |  |
| Total number regular medications  (categorised) | 617 | ≤ 2 | 75 (12%) |
|  |  | 3 – 5 | 308 (50%) |
|  |  | 6 – 10 | 208 (34%) |
|  |  | 11+ | 26 (4%) |
|  |  |  |  |
| Polypharmacy (5+ medications) | 617 | No | 383 (62%) |
|  |  | Yes | 234 (38%) |
|  |  |  |  |
| Anti-seizure medication (ASM) | 616 | 0 | 8 (1%) |
|  |  | 1 – 2 | 354 (57%) |
|  |  | 3 – 4 | 222 (36%) |
|  |  | 5+ | 32 (5%) |
|  |  |  |  |
| Vagal nerve stimulation (VNS) | 616 | No | 563 (91%) |
|  |  | Yes | 53 (9%) |
|  |  |  |  |
| Antipsychotics | 617 | 0 | 444 (72%) |
|  |  | 1 | 158 (26%) |
|  |  | 2+ | 15 (2%) |
|  |  |  |  |
| Other psychotropic medications | 616 | 0 | 417 (68%) |
|  |  | 1 | 172 (28%) |
|  |  | 2+ | 27 (4%) |
|  |  |  |  |
| PRN medications | 605 | 0 | 196 (32%) |
|  |  | 1 – 2 | 347 (57%) |
|  |  | 3+ | 62 (10%) |
|  |  |  |  |
| Physical side effects | 617 | No | 512 (83%) |
|  |  | Yes | 105 (17%) |
|  |  |  |  |
| Psychiatric side effects | 617 | No | 596 (97%) |
|  |  | Yes | 21 (3%) |
|  |  |  |  |

Summary statistics: number (percentage) or median [inter-quartile range]
